# Retraction: Evaluation of Live Recombinant Nonpathogenic *Leishmania tarentolae* Expressing Cysteine Proteinase and *A2* Genes as a Candidate Vaccine against Experimental Canine Visceral Leishmaniasis

**DOI:** 10.1371/journal.pone.0218050

**Published:** 2019-07-05

**Authors:** The PLOS ONE Editors

After publication of this article [1], it came to light that research ethics concerns that had been raised during the submission’s original peer review process had not been fully addressed prior to the article’s acceptance. These concerns included that the study used stray dogs without clear scientific or ethical justification, humane endpoints were not clearly reported for the survival study, and it was unclear from the published methods whether the health monitoring protocol and enrichment considerations were adequate given the nature of the disease under study and the animal model.

We discussed these issues with the authors who provided the following clarifications:

- Stray dogs were used for the study because there is no breeding center for lab dogs in Iran. Also, in designing the study the authors sought to test the vaccine candidate in a population heterogeneous for breed, genetic composition, diet, and microbiota so as to emulate the diverse population for which a successful vaccine would be utilized. The dogs were obtained from Tehran and Mashhad which the authors described as non-endemic parts of Iran, and they were not wearing identifying tags indicative of pet ownership.
- The experiment was designed with an endpoint of 20 months post-infection, and the following endpoint criteria were described in the approved study protocol: long term anorexia, hemoptesis, emaciation, reluctance to move.
- The two animals that died from the experimental infection without humane euthanasia did not show clear signs of illness before death.
- The authors observed some weight loss in the control group as compared to the vaccinated group.
- Three dogs were reported as having died from unrelated causes; authors noted that causes of death in these cases included gastric dilation and volvulus, uremia, and kidney disease. The authors were unable to clarify retrospectively whether these three animals were euthanized or died without intervention.
- Animals were monitored daily as to appetite and activity level, and thorough veterinary checks including physical examinations, body weight measurements, complete blood count (CBC), and serum biochemistry diagnostics were conducted once every three months.
- With regard to environmental enrichment, socialization, and exercise considerations, the dogs were provided commercial bones, were allowed to socialize within each group, were in contact with three people who fed animals and took them outside, and were afforded 30 minutes of outdoor access with other animals of their group each day.

As noted in *PLOS ONE*’s submission guidelines, articles reporting the use of random source animals and/or studies in which death is an experimental endpoint are subject to editorial scrutiny and reporting requirements (https://journals.plos.org/plosone/s/submission-guidelines#loc-animal-research). Animal research studies reported in *PLOS ONE* must adhere to internationally accepted research ethics standards, and we reserve the right to reject or retract work if such standards have not been met per our editorial assessment (https://journals.plos.org/plosone/s/animal-research).

In this case, we consulted *PLOS ONE*’s external Animal Research Advisory Group about the issues raised and the information provided post-publication by the authors. In light of the advice received from this Animal Research Advisory Group, the *PLOS ONE* Editors consider that this study did not meet the journal’s animal research standards due to the following concerns:

- Per our assessment and advice received from the Animal Research Advisory Group, there was not sufficient justification for the use of stray dogs in this study, and the use of stray dogs raises ethical concerns about the original sources of these animals and how they were obtained for research. Furthermore, the use of stray dogs presents scientific concerns with implications for the results and the generalizability of the conclusions, in that unknown confounds based on the animals’ health history and/or prior exposures may have impacted the outcomes of this study.
- The Animal Research Advisory Group advised that the record keeping, health monitoring protocols (including the nature and frequency of veterinary checks) and humane endpoint criteria used were not adequate given the disease model under study. Concerns were also noted about the socialization and activity regimens provided to the animals.
- The use of the control group and the sizes of control and experimental groups in the study were not adequately justified.

In light of these concerns about animal research ethics issues, welfare considerations, and the study design, the *PLOS ONE* Editors retract this article. We acknowledge that this decision reflects the journal’s research ethics standards but may not be representative of all institutions’ positions on the above issues. The study received approval from the Institutional Animal Care and Research Advisory Committee of Pasteur Institute of Iran and the Veterinary Board of Tehran Medical School.

We regret that these issues were not addressed in full prior to the article’s publication.

The authors disagree with the retraction.
